# Acidulated Tooth Powders

**Published:** 1851-04

**Authors:** 


*Acidulated Tooth Powders*—Our attention has been frequently called to advertisements of "tooth powders," for whitening, beautifying and preserving the teeth, and we have found that nearly all the dentifrices thus advertised, which we have examined, contained more or less tartaric acid, which, according to the tables of elective attraction, has a stronger affinity for the lime of the teeth than the phosphoric with which it is combined, and, consequently, must exert a most destructive action upon these organs. A powder having this acid for its base, will, for a time, unquestionably, "whiten" and "beautify" the teeth; but it will also, if used for a considerable length of time, as certainly cause their destruction and eventual discoloration. The sale of such an article can only be effected but for a short time, as their pernicious influences exhibit themselves, and, of course, are laid aside. Why a dentist should resort to such a composition, is, indeed, strange, as a most superior article can and is made free from such, and which will not only assist in cleansing the teeth, but preserves the health and tenacity of the gums, purify the breath, and add much to the general comfort of the mouth,

Spurious preparations, as "*tooth enamels,*" washes for removing tartar, &c., are most generally prepared by individuals unacquainted with the wants and character of a good powder, and whose only aim is to make money by a pretty exterior, regardless of the injury and destruction inflicted. Such as these are found in almost every shop window, and many druggists scruple not to vend an article which it is their duty to know, is most objectionable, greatly imposing upon the confidence of a confessedly ignorant public, by assurances unfounded and, in truth, unreasonable.

It is high time the profession should give some attention to this matter, and advise and instruct their patrons to avoid such detestable mixtures.

